# Chemotherapeutics Combined with Luminal Irritants: Effects on Small-Intestinal Mannitol Permeability and Villus Length in Rats

**DOI:** 10.3390/ijms23031021

**Published:** 2022-01-18

**Authors:** Maria-José Cano-Cebrián, David Dahlgren, Fredrik Kullenberg, Karsten Peters, Tobias Olander, Markus Sjöblom, Hans Lennernäs

**Affiliations:** 1 Department of Pharmacy, Pharmaceutical Technology and Parasitology, University of Valencia, 46100 Burjasot, Spain; Maria.Jose.Cano@uv.es; 2 Department of Pharmaceutical Biosciences, Translational Drug Discovery and Development, Uppsala University, 752 36 Uppsala, Sweden; david.dahlgren@farmbio.uu.se (D.D.); Fredrik.kullenberg@farmbio.uu.se (F.K.); Karsten.peters@farmbio.uu.se (K.P.); olander92@hotmail.com (T.O.); 3 Department of Medical Cell Biology, Division of Physiology, Uppsala University, 751 23 Uppsala, Sweden; markus.sjoblom@neuro.uu.se

**Keywords:** chemotherapy-induced mucositis, intestinal permeability, villus atrophy, cytostatics, toxicity, mannitol

## Abstract

Chemotherapy causes intestinal mucositis, which includes villous atrophy and altered mucosal barrier function. However, there is an uncertainty regarding how the reduced small-intestinal surface area affects the mucosal permeability of the small marker probe mannitol (MW 188), and how the mucosa responds to luminal irritants after chemotherapy. The aims in this study were to determine (i) the relationship between chemotherapy-induced villus atrophy and the intestinal permeability of mannitol and (ii) how the mucosa regulate this permeability in response to luminal ethanol and sodium dodecyl sulfate (SDS). This was investigated by treating rats with a single intraperitoneal dose of doxorubicin, irinotecan, or 5-fluorouracil. After 72 h, jejunum was single-pass perfused and mannitol permeability determined at baseline and after 15 min luminal exposure to 15% ethanol or 5 mg/mL SDS. Tissue samples for morphological analyses were sampled from the perfused segment. All three chemotherapeutics caused a similar 30% reduction in villus length. Mannitol permeability increased with irinotecan (1.3-fold) and 5-fluorouracil (2.5-fold) and was reduced with doxorubicin (0.5-fold), suggesting that it is not epithelial surface area alone that regulates intestinal permeability to mannitol. There was no additional increase in mannitol permeability induced by luminal ethanol or SDS in the chemotherapy-treated rats compared to controls, which may be related to the relatively high basal permeability of mannitol compared to other common low-permeability probes. We therefore suggest that future studies should focus on elucidating the complex interplay between chemotherapy in combination with luminal irritants on the intestinal permeability of other probes.

## 1. Introduction

Cancer is the second-largest cause of global premature death before the age of 70, and there is a significant medical need for effective and safe anticancer therapies that will prolong life [1]. Chemotherapeutics kill fast-growing and dividing cancer cells by unselectively inhibiting DNA replication, mitosis, or other important cell cycle functions. This means that proliferating healthy cells throughout the body are also affected. The intestinal mucosa is especially vulnerable because it is completely renewed about once per week by the rapidly dividing stem cells in the crypts of the epithelium [2]. The consequence is off-target gut toxicity, or chemotherapy-induced mucositis (CIM), which is a common condition in cancer patients dosed with anti-neoplastic drugs [3]. Clinically, it is primarily associated with crippling diarrhea [4], but anorexia, pain, nausea, and sepsis are also common.

In addition to these clinical manifestations, hallmarks of the condition are an impaired absorptive capacity, small intestinal villous atrophy (i.e., a reduction in total epithelial surface area [5]), a lower number of mature enterocytes, and altered intestinal barrier function [6]. In both humans and animal models, the degree of villous atrophy is determined from biopsies. Barrier function, however, is typically measured indirectly by monitoring the plasma exposure and/or renal clearance of non-metabolized paracellular marker probes following oral ingestion [7]. In CIM, the intestinal permeability of the low-molecular-mass probes is assumed to decrease as a result of a reduction in the total intestinal surface area, similar to what is observed with celiac disease [8,9]. However, to our knowledge, no direct measurement has been reported of in vivo intestinal permeability of low-molecular-mass probes following chemotherapy. A direct measurement from the intestines ignores confounding factors in the absorption process, such as gastric emptying, intestinal transit, distribution, and renal elimination. Direct measurements would better clarify the role of different chemotherapeutics in CIM [10] and the complex relationship between mucosal injury and barrier dysfunction in general [11].

Extensive mucosal injury and disease may render the intestinal barrier unrestrictive even to large bacteria [12]. However, under normal conditions, the small intestinal mucosa can handle everyday chemical and mechanical stressors from the luminal side, including high concentrations of bile salts, low pH, and various food components. It can also handle many luminal irritants that are not part of a normal diet, such as ethanol, an organic solvent with central stimulant effects, and sodium dodecyl sulfate (SDS), an anionic surfactant commonly used as a pharmaceutical excipient to optimize drug particle disintegration and dissolution in the GI tract. For instance, small intestinal permeability has been measured in the rat lumen exposed to 15% ethanol *w*/*w* [13], or to 5 mg/mL of SDS [14], which corresponds to the upper small intestinal concentrations following oral intake of 80 mL of 40% ethanol [15] or 1 g SDS [16], respectively. The permeability marker ^51^Cr-EDTA increased noticeably after the luminal exposures but returned to baseline within an hour. This illustrates the remarkable ability of the mucosa to respond to injury and uphold homeostasis even in challenging luminal conditions.

However, a prerequisite for the intestine to rapidly restore a compromised epithelium is a functional restitution process that fills epithelial cellular gaps [17]. An active enteric nervous system is also necessary, as is the normal expression and function of tight junction proteins, which regulate the paracellular transport [18]. Currently, it is not clear what impact chemotherapy has on the normal mucosal injury-response to luminal irritants. A better understanding of these chemotherapeutic effects and the adaptive processes involved in tissue repair is crucial for the development of treatments for CIM, a condition currently lacking effective intervention strategies [19].

The primary objectives of this study were to evaluate the effect of three chemotherapeutics on rat jejunal mucosal histology (villus length and crypt depth), permeability of a low-molecular-mass probe (mannitol), and the relationship between villus length and intestinal permeability. The secondary aim was to evaluate the ability of the jejunum to withstand ethanol- and SDS-induced increases in intestinal permeability following chemotherapy. This was performed by dosing rats with saline or with one of three chemotherapeutics, doxorubicin (DOX), irinotecan (IRI), or 5-fluorouracil (5-FU). After 72 h, when villus atrophy in rodents is reported to be at its greatest [20,21], the jejunum was single-pass perfused with a buffer solution followed by two luminal irritants: ethanol or SDS. During this time, the basal and irritant-induced increase in blood-to-lumen transport of mannitol was monitored. Intestinal samples for histological analysis were sampled adjacent to and inside the perfused segment to evaluate both the effect of chemotherapy alone and in combination with the luminal irritants.

## 2. Results

### 2.1. Basal Intestinal Permeability

**Figure 1:**
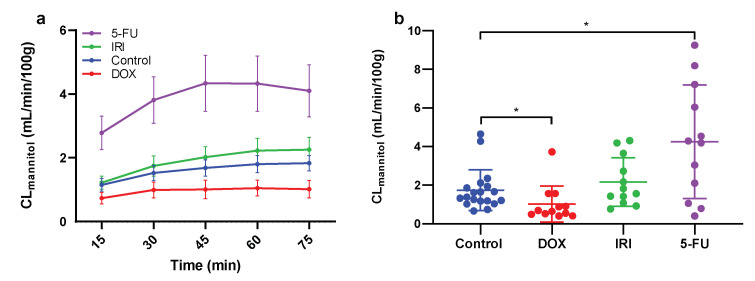
The experiment was performed 72 h after the rats were dosed with saline (control), or with either doxorubicin (DOX), irinotecan (IRI), or 5-fluorouracil (5-FU). (**a**) The mean (±SEM) blood-to-lumen clearance of mannitol (CL_mannitol_) during single-pass perfusion of the jejunum with a buffer solution during the first 75 min of the perfusion experiment. The average CL_mannitol_ at steady state (between 45 and 75 min) was regarded as representative for each rat. (**b**) The mean (±SD) and individual CL_mannitol_ values after the different treatments. A * represents a significant difference in CL_mannitol_ between the controls and the chemotherapy-treated rats.

The mean (±SEM) baseline CL_mannitol_ over time (the first 0–75 min in each group) for the control (*n* = 18), and DOX, IRI, and 5-FU groups (*n* = 12 each) are presented in Figure 1a. The mean (±SD) CL_mannitol_ for each rat at steady-state (30–75 min) was used to compare the difference in baseline intestinal permeability between the control and the three chemotherapy groups (Figure 1b). The DOX group had a significantly lower jejunal CL_mannitol_ (1.02 ± 0.94 g mL/min/100 g) compared to the control (1.74 ± 1.06 mL/min/100 g). Conversely, the jejunal CL_mannitol_ was significantly (*p* < 0.05) higher for 5-FU (4.26 ± 2.95 mL/min/100 g). The value for IRI, while higher, was not statistically significant (2.17 ± 1.26 mL/min/100 g).

### 2.2. Villi Length and Crypt Depth

**Figure 2:**
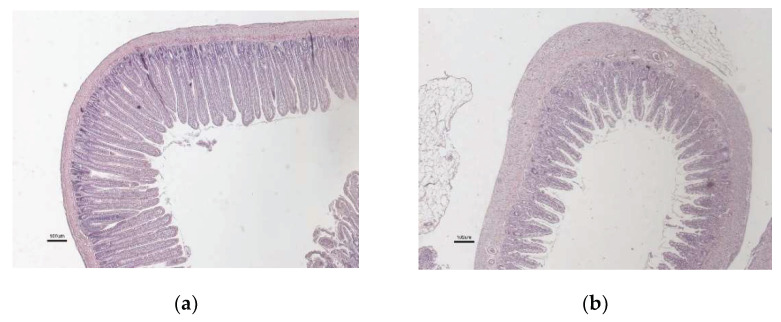
Jejunal mucosa for the (**a**) healthy control rats and (**b**) at 72 h after chemotherapy (there were no obvious differences between DOX, IRI, and 5-FU). Scale bar = 100 µm.

Typical rat jejunal mucosa at control conditions, and at 72 h after chemotherapy, are shown in Figure 2a,b, respectively (only one histological picture following chemotherapy is shown as there were no obvious differences between DOX, IRI, and 5-FU.) 

**Figure 3:**
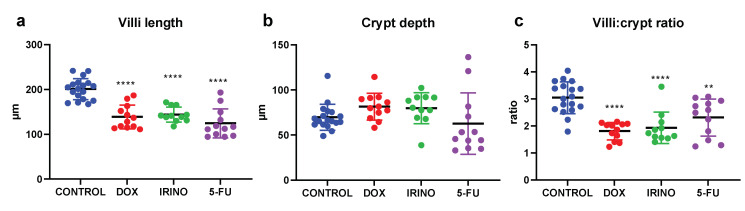
The mean (±SD) and individual (**a**) villus length, (**b**) crypt depth, and (**c**) villi:crypt ratio measured 72 h after the rats were dosed with saline (control), or with either doxorubicin (DOX), irinotecan (IRI), or 5-fluorouracil (5-FU). ** *p* < 0.005 and **** *p* < 0.00005 represent statistically significant differences compared to the control rats.

Figure 3a–c shows the mean (±SD) jejunal villi length, crypt depth, and villi:crypt ratio for the control rats and 72 h after dosing with DOX, IRI, or 5-FU. The villi length decreased significantly (*p* < 0.05) compared to the control (201 ± 24 µM) for all three chemotherapy groups: DOX (139 ± 27 µM), IRI (144 ± 17 µM), and 5-FU (124 ± 32 µM). For the crypt depth, there were no significant differences for any of the chemotherapies compared to the control group (70 ± 14 µm), but there was a slight increase for DOX (82 ± 15 µm) and IRI (80 ± 17 µm), and a small decrease for 5-FU (63 ± 34 µm). Combining the two morphological measurements (i.e., villi:crypt ratio) gave a significantly (*p* < 0.05) lower ratio compared to the control (3.0 ± 0.6): DOX (1.8 ± 0.3), IRI (1.9 ± 0.6), and 5-FU (2.3 ± 0.7).

### 2.3. Correlation between Basal Intestinal Permeability and Villi Length

**Figure 4:**
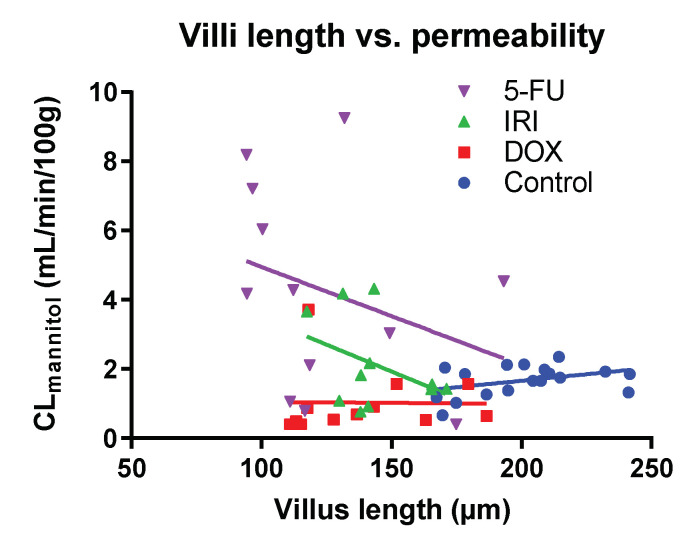
Correlation between villus length and blood-to-lumen clearance of mannitol (CL_mannitol_) in each rat. The determinations were performed 72 h after the rats were dosed with saline (control), or with either doxorubicin (DOX), irinotecan (IRI), or 5-fluorouracil (5-FU).

Figure 4 shows there was no correlation between villus length (corresponding to surface area) and basal CL_mannitol_ (intestinal permeability) for the combined dataset (R^2^ = 0.1), or for any of the four individual groups: control (R^2^ = 0.16), DOX (R^2^ < 0.01), IRI (R^2^ = 0.16), or 5-FU (R^2^ = 0.10).

### 2.4. Effect of Luminal Irritants on Intestinal Permeability and Histology

**Figure 5:**
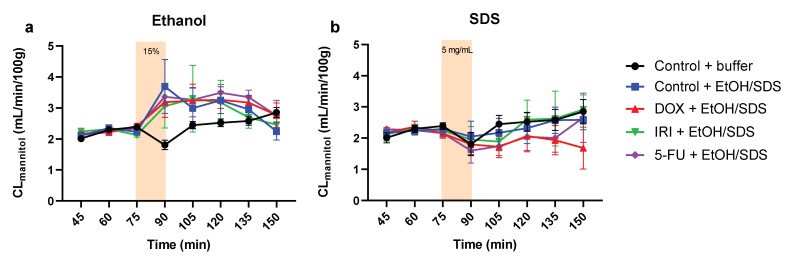
The mean (±SEM) blood-to-lumen clearance of mannitol (CL_mannitol_) during single-pass perfusion of the jejunum with a buffer solution for 75 min, followed by a 15 min perfusion with (**a**) 15% ethanol (EtOH) or (**b**) 5 mg/mL sodium dodecyl sulfate (SDS), followed again with a 60 min perfusion with buffer. The ethanol and SDS perfusions were performed on control rats (blue), and on rats 72 h after treatment with doxorubicin (DOX, red), irinotecan (IRI, green), and 5-fluorouracil (5-FU, purple). The control rats were also perfused with only buffer during 150 min in one group (black).

The mean (±SEM) CL_mannitol_ values over time (0–150 min) in the control and chemotherapy-treated rats are presented in Figure 5a (ethanol) and Figure 5b (SDS). These irritants were added to the lumen between 75 and 90 min. Ethanol induced a significant (*p* < 0.05), and similar (about twofold), increase in CL_mannitol_ at the end of the 15 min luminal exposure (at 90 min) for all four treatment groups (control, DOX, IRI, and 5-FU) compared to control rats perfused only with a buffer solution for 150 min. SDS induced no increase in CL_mannitol_ compared to the buffer solution for any of the four treatment groups.

**Figure 6:**
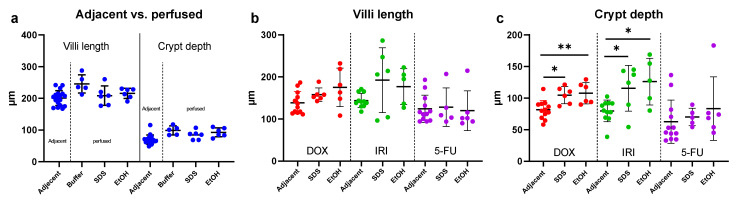
(**a**) Villi length and crypt depth in the non-chemotherapy treated control rats. (**b**) Villi length and (**c**) crypt depth, 72 after treatment with doxorubicin (DOX), irinotecan (IRI) and 5-fluorouracil (5-FU). The adjacent histological segment was sampled outside of the perfused intestinal segment, whereas the other histological segments were sampled 60 min after perfusion with buffer, 15% ethanol (EtOH), or 5 mg/mL sodium dodecyl sulfate (SDS). Data presented as mean (±SD). * *p* < 0.05 and ** *p* < 0.005 represent statistically significant differences between the adjacent and perfused segments.

Intestinal segments perfused with buffer, SDS, or ethanol were analyzed for villi length and crypt depth. In control animals, there was no impact on either villus length or crypt depth from perfusing the segment with a buffer compared to the samples taken adjacent to the perfused segment (Figure 6a). Likewise, there were no differences in villus length or crypt depth induced by SDS or ethanol compared to the buffer. For the chemotherapy-treated rats, there was no effect on villus length of perfusing the intestine with either SDS or ethanol, compared to the adjacent intestinal segment in the same animal (Figure 6b). However, both SDS and ethanol significantly (*p* < 0.05) increased the crypt depth in the DOX- and IRI-treated rats, compared to the adjacent intestinal segment in the same animal (Figure 6c).

## 3. Discussion

This study primarily aimed to elucidate the relationship between chemotherapy-induced villus atrophy and intestinal mucosal mannitol permeability. Secondly, it aimed to examine the ability of the intestinal mucosa to regulate intestinal mannitol permeability in response to luminal irritants after chemotherapy. This was investigated by treating rats with three different classes of chemotherapeutics: DOX, primarily a topoisomerase II inhibitor; IRI, a topoisomerase I inhibitor; and 5-FU, an antimetabolite. After 72 h, the intestine was single-pass perfused during which the permeability of mannitol was examined before, during, and after luminal exposure to ethanol or SDS. Jejunal tissues for morphological analysis were sampled from both the inside of, and adjacent to, the perfused segment in each rat.

Anti-neoplastic drug therapy is notorious for its severe side effects, in particular chemotherapy-induced intestinal mucositis (CIM). CIM is characterized by morphological changes, such as villus atrophy, together with clinical symptoms of diarrhea, pain, malnutrition, bacterial translocation, and sepsis. The latter two result from therapy-induced immunosuppression in combination with a compromised mucosal barrier. The epithelial barrier status is typically evaluated by measuring the intestinal permeability, which is defined as the ability of the intestine to resist passive transport of water-soluble probes of different sizes and charges. Experimentally, in vivo permeability is usually determined *indirectly* from radioactive or fluorescent probes in plasma or urine following oral dosing [7]. An ideal probe molecule is inert and passively transported in the paracellular pores between cells, which are regulated by tight-junction proteins. However, following oral dosing, the rate and fraction absorbed of a probe also depends on non-permeability factors, such as the gastric emptying rate and intestinal transit time. Both factors can be extensively affected during CIM due to diarrhea. To compensate for these non-permeability factors, probes with different molecular sizes and charges are often combined. Thus, an increased permeability ratio (i.e., large vs. small probe) reflects a reduced surface area and/or increased paracellular leakage [22]. This is based on the assumption that somewhat larger probes, such as ^51^Cr-EDTA (340 Da) and lactulose (342 Da), are transported only in the leaky pores in the crypt region. The smaller probes, such as mannitol (182 Da), are assumed to be transported paracellularly in the less leaky pores residing over the whole crypt–villus axis [23].

However, the assumption that the surface area reflects epithelial mass transport of mannitol has not been verified in any *direct* determinations of intestinal permeability in relation to CIM. A preclinical method well suited for this evaluation is the SPIP model [24,25]. It relies on transepithelial solute flux at controlled in vivo conditions, in which the intestinal permeability is maintained by hormonal, neural, and paracrine regulation. As such, the SPIP model is often used to investigate intestinal physiology, solute transport, and mucosal injury [26,27]. As rodents are commonly used for evaluating intestinal toxicity of chemotherapy [28], we believe that the rat SPIP model is ideal for evaluating alterations in intestinal permeability in CIM, with or without the presence of luminal irritants.

Following chemotherapy to rodents, maximum histological injury occurs after about three days [20,21]. In our study, all the chemotherapies (DOX, IRI, and 5-FU) decreased the villus length by ≈30% after 72 h, in good agreement with similar studies [29]. Based on the assumption that the primary determinant of intestinal permeability of mannitol is the epithelial surface area available for transport, we expected to see a reduced permeability for all three drugs. Indeed, this was the case for DOX, for which there was a 41% reduction in basal CL_mannitol_. However, for IRI, there was no statistically significant difference compared to the control animals (25% increase), while for 5-FU, basal CL_mannitol_ increased 150%. Furthermore, we were unable to detect any correlation between villus length and CL_mannitol_ for any of the chemotherapy drugs individually, or for the combined dataset. This contradicts the mannitol permeability vs. surface-area paradigm and suggests that other changes important for the intestinal permeability of mannitol are involved. Probably the epithelial leakiness of mannitol increases, at least for 5-FU and IRI. This may be via alteration of the integrity of the epithelial cell monolayer and/or the paracellular junction proteins, as the latter are known to be regulated by proinflammatory cytokines, such as TNFα and IL-4, in the intestines [30]. Thus, it seems that the permeability effects are related to the individual cytokine profile generated by the different classes of chemotherapeutic drugs used in this study. For instance, IRI and 5-FU generate a strong increase in the proinflammatory cytokines TNFα and IL-1β [31,32]. In contrast, DOX has no effect on TNFα but increases IL-6 sharply [33]. Following 5-FU dosing in mice, a possible relationship is also seen between an increased intestinal permeability of 99mTc-DTPA (MW: 487 Da) and increased expression of TNFα and IL-6 [34]. In summary, our novel *direct* permeability data have implications for the choice of probe, and interpretation of results. This applies to results for permeability studies in general, and for CIM in particular. A better understanding is needed for how different chemotherapeutics and their associated inflammatory and secretory responses affect the intestinal permeability of different probes. This is especially true if intestinal permeability is used as a surrogate endpoint in evaluating the effect of CIM interventions [35].

Our results show that different chemotherapeutics give rise to similar morphological changes. These, in turn, can have substantially different effects on intestinal functions, including membrane transport. The increase in CL_mannitol_ was greatest for 5-FU followed by IRI. Clinically, these are also the two chemotherapeutics that cause the most severe GI disorders [36], especially in combination with each other, causing severe diarrhea in up to 80% of the patients [4]. If these two CIM parameters—increased CL_mannitol_ and diarrhea—are related to each other, the mucosal barrier could be targeted [37] in the treatment of chemotherapy-induced late-onset diarrhea.

Apart from the dysregulation as a result of chemotherapeutics, chronic intestinal barrier dysregulation is seen in many GI and systemic diseases and disorders, such as celiac and inflammatory bowel disease, obesity and type 1 diabetes, non-alcoholic fatty- and alcoholic-liver disease, and irritable bowel syndrome [38]. A healthy intestine forms a selective barrier that balances optimal protection against harmful luminal microorganisms and proteins/xenobiotics/toxins while allowing efficient nutrient absorption. The intestine has a key ability to rapidly respond to, and heal, any injury from luminal irritants and mechanical stress. These irritants may be endogenous, including bile acids from the pancreas or the high acidic load (pH < 2) from the stomach, or originate from oral intake. Two examples from the latter category are ethanol and SDS. Ethanol is an organic solvent with central stimulant effects, and SDS is an anionic surfactant commonly used as a pharmaceutical excipient to optimize drug particle disintegration and dissolution in the GI tract. Both compounds cause local mucosal injury as well as inducing substantial increases in epithelial permeability at physiologically relevant exposure times [18,39]. In a healthy intestine, these changes are repaired within an hour through a functioning restitution process, in combination with a neural and paracrine response system that maintains mucosal (and systemic) homeostasis [3]. There is, however, uncertainty regarding the capability of a chemotherapy-compromised intestine to deal with, and respond to, luminal irritants that normally cause only transient effects.

In this study, ethanol induced a similar twofold increase in CL_mannitol_ in both the control animals and chemotherapy-treated ones, suggesting that anti-neoplastic drug treatment had no impact under the conditions investigated. However, similar to the conclusions from the basal permeability data in this study, the results may be related to the choice of probe. The epithelial membrane may not have sufficiently restricted mannitol transport, thereby making it impossible to detect any difference between the chemotherapy and control groups. For instance, ^51^Cr-EDTA and inulin are two larger paracellular probes primarily transported in the crypt regions rather than over the whole villus such as mannitol. Both ^51^Cr-EDTA and inulin have a 5- to 20-fold lower basal permeability in the rat upper small intestine and may thus respond differently than mannitol [23,40]. Indeed, we saw no SDS-induced increase in mannitol permeability in any of the treatment groups, despite the numerous reports of its positive effect on the intestinal permeability of ^51^Cr-EDTA [18,41].

Even if no significant differences in CL_mannitol_ were detected between the chemotherapy and control groups, the crypt depth increased slightly in the intestinal segment perfused with ethanol and SDS compared to buffer 72 h after dosing with DOX and IRI. The rapid onset of this increase in crypt depth (150 min) is probably unrelated to the compensatory crypt hyperplasia that is observed about 3–5 days after high doses of stem-cell apoptotic chemotherapy [42]. A more likely explanation is that the luminal irritants trigger mucosal edema. Why this effect was only observed for DOX- and IRI-treated rats, but not the control and 5-FU ones, remains to be investigated. If this increased sensitivity contributes to the mucosal inflammation in CIM, anti-inflammatory treatment is a possible option for CIM interventions.

In conclusion, this study showed that the epithelial surface area was not the only parameter determining the effect of chemotherapy on the intestinal permeability of mannitol. The increase in permeability with IRI and 5-FU, compared to DOX, may explain the severe and frequent diarrhea seen clinically with the former two drugs. In addition, there was no additional increase in mannitol permeability induced by luminal ethanol and SDS in chemotherapy-treated rats compared to the controls. This may be related to the relatively high basal permeability of mannitol compared to other commonly used permeability probes. We therefore suggest that future studies elucidate the complex interplay between different chemotherapeutics and doses in combination with luminal irritants on the intestinal permeability of different probes. Furthermore, an investigation is required to explain the mechanisms behind the increased crypt depth in DOX and IRI-treated rats after luminal exposure to SDS and ethanol.

## 4. Materials and Methods

### 4.1. Chemicals

Atropine, Accustain^®^ formalin solution (10%, neutral buffered), dimethyl sulfoxide, ethanol, Inactin (thiobutabarbital sodium), SDS, and bovine albumin were purchased from Sigma-Aldrich (St. Louis, MO, USA). Sodium phosphate dibasic dihydrate (Na_2_HPO_4_·2H_2_O), potassium dihydrogen phosphate (KH_2_PO_4_), sodium hydroxide (NaOH), and sodium chloride (NaCl) were purchased from Merck KGaA (Darmstadt, Germany). 5-Fluorouracil Teva (solution for injection, 50 mg/mL), Irinotecan Actavis (solution for infusion, 20 mg/mL), and Dynastat (parecoxib, powder for solution for injection, 40 mg) were purchased from Apoteket AB (Sweden). Doxorubicin hydrochloride was purchased from Toronto Research Chemicals (Toronto, ON, Canada). ^3^H-Mannitol was purchased from PerkinElmer Life Sciences (Boston, MA, USA).

### 4.2. Study Formulations

Three chemotherapeutics were used in this study. 5-FU (solution, 50 mg/mL) and IRI (solution, 20 mg/mL) were obtained in ready-to-use form, whereas a 100 mM stock solution of DOX hydrochloride in dimethyl sulfoxide was prepared and diluted to 5 mg/mL in saline on the day of drug administration (final dimethyl sulfoxide concentration < 5%). Parecoxib (10 mg/mL), inactin (50 mg/mL), and atropine (0.1 mg/mL) were dissolved in saline and used within the recommended stability time. For the single-pass intestinal perfusion (SPIP) experiments, three isotonic (290 mOsm) phosphate buffer (8 mM) perfusate solutions were prepared at pH 6.5. The control solution contained only buffer, and two test buffer solutions contained SDS at 5 mg/mL (17.3 mM) or 15% *w*/*w* ethanol. With the exception of the ethanol solution, osmolarity was determined (after addition of all perfusate constituents) by freeze-point depression using a micro osmometer (Model 3MO; Advanced Instruments, Needham Heights, MA, USA).

### 4.3. Animals

This rat study was approved by the local ethics committee for animal research (5.8.18-17754/2019) in Uppsala, Sweden. Rats were male Wistar Han IGS from Charles River Co. (France) weighing 340 ± 100 g. They were delivered to the animal laboratory facility in Uppsala, Sweden at least one week before the experiment. Before and during the experiments, the rats were kept in enriched cages with free access to food and water on a 12:12 h light–dark cycle, 21–22 °C.

### 4.4. Chemotherapy Dosing and Intestinal Perfusions

At 72 ± 2 h before the SPIP permeability experiments, the rats were divided into four treatment groups (Table 1). The control group (*n* = 18) received an intraperitoneal injection of saline, and the three chemotherapy groups (*n* = 12 in each) received a single intraperitoneal injection of either DOX (10 mg/kg, 0.4–0.7 mL), 5-FU (200 mg/kg, 1–2 mL), or IRI (150 mg/kg, 2–3 mL). Immediately before the dosing with IRI, the rats also received a subcutaneous injection with atropine (0.02 mg/rat) to avoid acute and transient cholinergic side effects of this cytostatic prodrug [43,44].

The surgical procedure and experimental setup of the rat SPIP experiment has been previously described [45]. In short, the rats were anesthetized on the study day using an intraperitoneal injection of a 5% *w*/*v* inactin solution (180 mg/kg). The body temperature was maintained at 37.5 ± 0.5 °C. The systemic arterial blood pressure was continuously recorded to validate the condition of the animal. This was performed by connecting an arterial catheter to a transducer operating a PowerLab system (AD Instruments, Hastings, UK). Rats with a mean blood pressure below 70 mmHg were excluded from the study. For the SPIP experiment, the abdomen was opened along the midline and a 10–12 cm jejunal segment was cannulated, covered with polyethylene wrap, and placed outside the abdomen [45].

**Figure 7:**
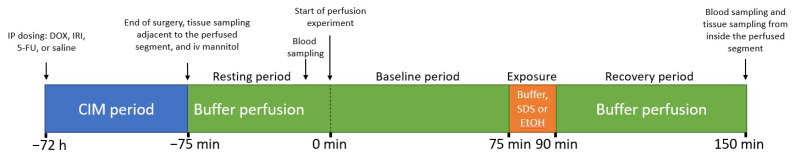
Study design, showing the 72 h chemotherapeutics-induced mucositis (CIM) period (Table 1) and the 225 min single-pass perfusion period (75 min resting and 150 min experiment). The intestinal mannitol permeability was monitored during the whole 150 min experimental period. DOX- doxorubicin, 5-FU—5-Fluorouracil, IRI—irinotecan, SDS—sodium dodecyl sulfate, EtOH—Ethanol.

The perfusion study design is illustrated in Figure 7. After completion of surgery, ^3^H-mannitol was administered intravenously as a bolus of 0.25 µCi (0.1 mL), followed by continuous intravenous infusion at 0.5 µCi per hour (1 mL/h) for the duration of the SPIP experiment. The first 60 min following surgery was a resting period, during which each intestinal segment was perfused with the control buffer solution. This stabilized cardiovascular, respiratory, and intestinal functions, and the ^3^H-mannitol levels in the plasma.

Following the 60 min resting period, three different SPIP experiments were performed while continuously monitoring the intestinal mannitol permeability (Figure 7). In one SPIP experiment, containing 6 of the 18 control rats, the control buffer solution was perfused during 120 min. The other two SPIP experiments were divided into three periods, in which both of them contained six rats from each of the four treatment arms (control, DOX, IRI, and 5-FU; see Table 1). Initially, (i) the jejunal segment was perfused with the control solution for 60 min to establish baseline permeability in each rat. This was directly followed by (ii) a 15 min perfusion with 5 mg/mL SDS or 15% ethanol to evaluate their effect on mucosal permeability and histology. Finally, (iii) the control buffer solution was again perfused for 60 min, during which time the recovery of the mucosa was evaluated. This experimental setup allowed us to evaluate the effect of chemotherapy on basal mucosal mannitol permeability (0–75 min), as well as the ability of the jejunal mucosa to respond to luminal irritants 72 h after chemotherapy (75–150 min).

All experimental periods started with a rapid filling (<20 s) of the entire jejunal segment with the perfusate (about 1.5 mL for a 10 cm segment). Thereafter, the single-pass perfusion rate was always 0.2 mL/min (peristaltic pump, Gilson Minipuls 3, Le Bel, France). The jejunal segment and perfusates were kept at 37 °C, and all outgoing perfusate was collected and weighed at 15 min intervals. The length and wet tissue weight of each jejunal segment was determined after the experiment. Blood samples (<0.3 mL) were drawn from the femoral artery before the start (t = 0 min) and then again at the end (t = 150 min) of the perfusion experiment. The blood samples were centrifuged (5000× *g*, 3 min at 4 °C) within 10 min, and the plasma was analyzed for ^3^H activity.

### 4.5. Determination of Blood-to-Lumen Jejunal ^3^H-Mannitol Clearance

In the SPIP experiments, 2 mL scintillation fluid (Pico-Fluor Plus, Perkin Elmer Life Sciences, Boston, MA, USA) was added to the luminal perfusates (1 mL), and the 0 and 150 min plasma samples (0.05 mL plasma with 0.95 mL water). These samples were then analyzed for ^3^H-mannitol in a scintillator (Tri-Carb 2910 TR, Perkin Elmer Life Sciences, Boston, MA, USA). Linear regression analysis of the plasma samples was made to obtain a corresponding plasma value for each perfusate sample. The blood-to-lumen mannitol clearance (CL_mannitol_) was calculated using Equation (1) [16].

(1) $${\mathrm{CL}}_{\mathrm{mannitol}}=\frac{C_{\mathrm{perfusate}\;}\times {\;Q}_{\mathrm{in}}}{C_{\mathrm{plasma}\;}\times \;\mathrm{tissue}\;\mathrm{weight}\;}\times 100$$

C_perfusate_ and C_plasma_ is the activity in the perfusate and plasma per mL, and Q_in_ is the flow rate in mL per min. In each group, CL_mannitol_ values over time (0 to 150 min) are calculated from the perfusion experiments: 0–75 min are the baseline mannitol permeability, and 75–150 min are the effects of the luminal irritants on mannitol permeability relative to the baseline period.

### 4.6. Histology

Two jejunal samples from each rat were collected for histology. One sample, adjacent to the perfused segment, was excised before the experiment. The other was excised from within the perfused segment, immediately after the end of the perfusion. These samples allowed us to evaluate the histological effects of chemotherapeutics, with and without luminal irritants (control, SDS and ethanol). The tissue samples were rinsed with saline and fixed in 10% formaldehyde for 24 h then transferred to 70% ethanol. They were then embedded in paraffin and microtome-sliced (Microm Cool-Cut HM 355 S, Thermo Fisher Scientific, Waltham, MA, USA) at 8 µm and dried overnight. Sections were de-paraffinized and rehydrated prior to staining. Hematoxylin-eosin staining was carried out according to standard practice [46].

**Figure 8:**
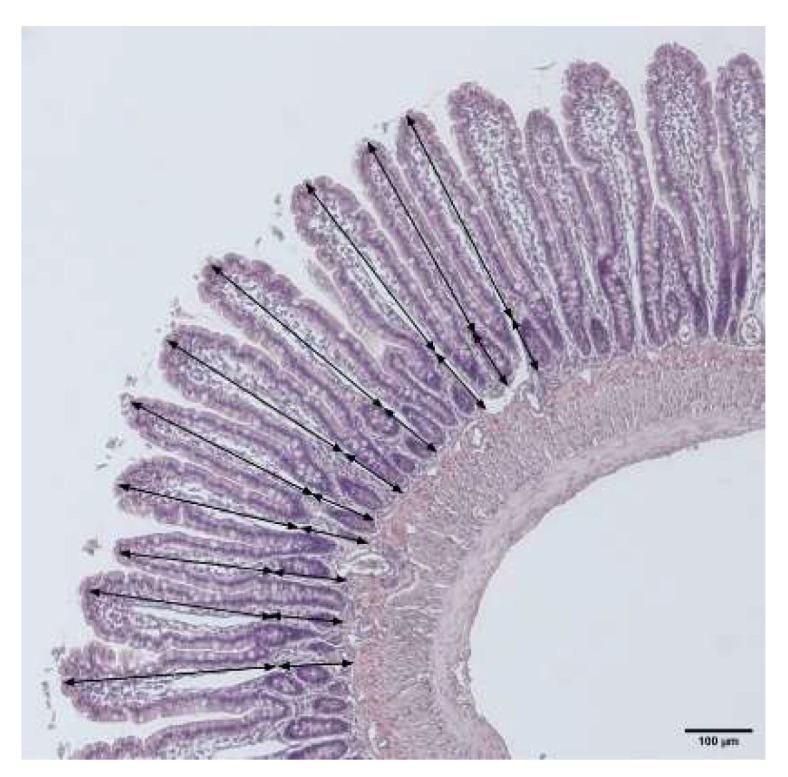
Method by which the villus length (finger-like protrusions) and crypt depth (area between villus bottom and muscle layer) were measured in each rat. The arrows show the length of each villus and crypt. The average of ten measurements was used for each animal.

The histological samples were analyzed for villi length and crypt depth, which were taken as measures of mucositis. Ten villi and crypts were measured (Figure 8) for each rat, and the mean was chosen as representative for that animal. Images were taken using an inverted confocal microscope (Eclipse TE2000-U, Nikon, Tokyo, Japan). The measurements were performed using the corresponding plugin in the ImageJ software version 1.50e (NIH, Bethesda, Maryland, USA).

### 4.7. Statistics

All data are expressed as mean ± SD or SEM. The villi length, crypt depth, villi:crypt ratio, basal CL_mannitol_, and CL_mannitol_ were analyzed directly after the end of the perfusion with luminal ethanol and SDS (t = 90 min). Rats dosed with DOX, IRI, and 5-FU were compared against the controls using a one-way, unpaired ANOVA, with a post hoc Dunnett’s comparison test. A *p*-value < 0.05 was regarded as significant in all analyses. Statistic tests and graphs were made in GraphPad Prism 9.1.2 (La Jolla, CA, USA).

## Figures and Tables

**Table 1:** The four experimental groups according to the treatment received. See Figure 7 for details of the perfusion. SPIP—single-pass intestinal perfusion; SDS—sodium dodecyl sulfate.

Intraperitoneal Dosing 72 h before SPIP	SPIP Exposure Period (15 min)
Saline, control (*n* = 18)	Buffer (*n* = 6)
	SDS 5 mg/mL (*n* = 6)
	Ethanol 15% *w*/*w* (*n* = 6)
Doxorubicin 10 mg/kg (*n* = 12) or Irinotecan 150 mg/kg (*n* = 12) or 5-Fluorouracil 200 mg/kg (*n* = 12)	SDS 5 mg/mL (*n* = 6)
	Ethanol 15% *w*/*w* (*n* = 6)

## Data Availability

The data presented in this study are available on request from the corresponding author.
